# The Impact of Virtual Interactive Three-Dimensional Model in the Conceptualization of the Anatomy of the Sacrum: A Randomized Controlled Trial

**DOI:** 10.7759/cureus.41514

**Published:** 2023-07-07

**Authors:** Pradip R Chauhan, Simmi Mehra, Ashish M Pandya

**Affiliations:** 1 Anatomy, All India Institute of Medical Sciences, Rajkot, IND; 2 Anatomy, Pandit Deendayal Upadhyay (PDU) Government Medical College, Rajkot, IND

**Keywords:** virtual three-dimensional models, ala of the sacrum, sacrum, anatomy, virtual reality, interactive 3d models

## Abstract

Introduction

Virtual interactive three-dimensional model (VI3DM) is an emerging technology with promising futures in medical education. It allows learners to view and interact with the three-dimensional (3D) object in an isolated virtual environment, as well as on screen-based platforms. This technology seems more helpful in understanding the learning objectives that demand high cognitive and visuospatial skills. The sacrum, part of the posterior wall of the bony pelvis, is a structure of interest to medical professionals and forensic experts. Understanding the anatomy and relations of the sacrum demands good spatial understanding. Hypothetically, virtual 3D models should help in learning the anatomy of the sacrum along with its relations and attachments. This study was conducted to find out the effect of low-cost digital 3D models on the anatomical knowledge of the study.

Aims and objectives

The goal of the work was to identify the role of virtual 3D models in the conceptualization of the anatomy of the sacrum. The study’s objectives were to identify the impact of virtual 3D models on students’ knowledge of the external features, relations, attachments, and joints formed by the sacrum.

Material and methods

Two hundred first-year medical students (168 males and 32 females) who participated in the study after providing informed consent were divided into two equal groups, a control group (n=100) and an experimental group (n=100), after matching the age, gender, and anatomical knowledge of the sacrum. We used two-dimensional (2D) images and virtual interactive 3D models of the sacrum as control and intervention, respectively, in this randomized controlled study. We conducted a post-test quiz after the 30-minute session of self-directed learning.

Results

The mean difference between the post-test score and the pre-test score of the experimental group (4.1±1.6 ) was significantly higher than the difference between the post-test and pre-test scores of the control group (2.5±1.2). The virtual interactive 3D model of the sacrum was significantly effective in the conceptualization of the sacrum anatomy.

Conclusion

A virtual interactive 3D model is an effective tool to conceptualize the anatomy of the sacrum and can be explored for its use in further complex anatomical structures. Digital 3D models can become a platform for the application of various virtual realities (VR) and artificial intelligences in medical education.

## Introduction

Medical education is a complex and vast curriculum designed to train a medical student to manage a patient independently [1,2]. As per the recommendations of the National Medical Commission, an Indian medical graduate (IMG) is supposed to acquire knowledge and skills of specific learning objectives [1,2]. Various medical teaching aids (e.g., a chalkboard, PowerPoint, and museum specimen) are used for the training of medical professionals [2]. Medical professionals are supposed to have hands-on experience with the procedures and skills along with in-depth theoretical knowledge [2,3]. In anatomy, spatial visualization is very important to conceptualize the relationships of any anatomical structure; that can be achieved when students perform any activity or interact with the learning materials [4,5]. In the Covid era, offline anatomical education, where students are generally trained with real human body parts and bones, was affected [6]. Plenty of videos and digital material are available on various online platforms, but the material is either not interactive or costly [5,6]. In this situation, various fields including medical sciences have been exploring the use of digital three-dimensional (3D) models, virtual interactive 3D models (VI3DM), augmented reality (AR), mixed reality (MR), and virtual reality (VR) in the last few years [7]. The 3D anatomy model is defined as follows: “Three-dimensional (3D) anatomy models comprise digital and non-digital (physical) models that can be moved into different positions/planes to enable the learner to learn the relationship between different anatomical structures in space and mentally manipulate objectives in three dimensions” [8]. Virtual or digital 3D models can be displayed on web browsers, computer screens, mobile screens, or mobile applications. The 3D models can be labeled to make them more interactive and represent anatomical facts [7,8]. According to previous studies in various fields, VI3DM is engaging and increases the interest of students in learning [5-9]. The sacrum has two surfaces, two borders, a base with important relations, and an apex articulating with the coccyx [10]. Hypothetically, VI3DM should effectively help to conceptualize the bony structure such as the sacrum, but previous studies have contradictory reports regarding the effect of VI3DM on students’ anatomical knowledge in comparison to conventional learning resources [11-17]. We conducted this study to test the following hypothesis: “The virtual interactive three-dimensional model helps to conceptualize the anatomy of the sacrum.”

## Materials and methods

This randomized controlled trial (parallel group) was approved by the Institutional Ethics Committee of All India Institute of Medical Sciences (AIIMS) (AIIMS RAJKOT/IEC/02/2021; project code: F-IM 01/2021) and conducted as per the guidelines of the World Medical Association Declaration of Helsinki “Ethical Principles for Medical Research Involving Human Subjects” at the Department of Anatomy at AIIMS, Rajkot, Gujarat, India. This multicenter interventional trial was registered in the Clinical Trial Registry of India (registration number: CTRI/2021/10/037621). VI3DM (learning resources) was used as interventional learning resources and two-dimensional (2D) images as control learning resources.

Preparation of learning material

**Figure 1:**
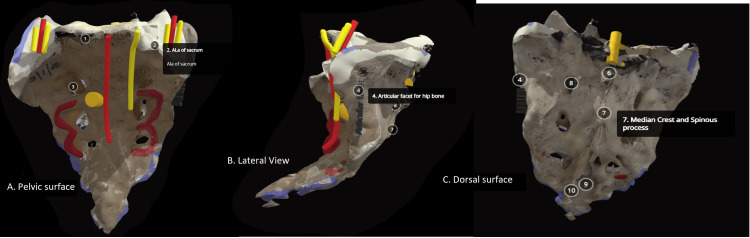
Screenshot of the virtual interactive 3D model of the sacrum: A, pelvic surface; B, lateral view; C, dorsal surface The virtual interactive 3D model of the sacrum is accessible from Sketchfab through the following link: https://sketchfab.com/3d-models/sacrum-e17065516e6a4573bac99ffca17f6944. The model was created by the author himself and uploaded on Sketchfab, available as open-access resource (Creative Commons License 4.0) 1, sacral promontory; 2, ala of the sacrum; 3, ventral sacral foramina; 4, articular facet for the ilium; 5, sacral canal; 6, spinous process; 7, median crest and spinous process; 8, posterior sacral foramen; 9, sacral hiatus; and 10, sacral cornu 3D: three-dimensional

The specimen of the sacrum used in the study was retrieved from the dry original bones available in the department. We photographed the sacrum with a high-definition digital camera and labeled the sacrum for the creation of 2D images. We used the same specimen to create a virtual 3D model (Figure 1 and Video 1). With the help of a licensed version of the Qlone application (EyeCue Vision Technologies Ltd., Yokneam, Israel), we uploaded it to the web-based Sketchfab application (Sketchfab, New York, NY) and labeled it.

**Video 1:** Interaction and demonstration of virtual interactive 3D model of the sacrum 3D: three-dimensional

We used the 2D images to create conventional learning material and an interactive 3D model for the interventional learning material.

Participants

The sample size was calculated using the formula for the superiority parallel group randomized controlled trial: $Sample size= 2s^{2}\frac{(z_{1-\alpha /2}+z_{1-\beta })}{\delta^2 }^{2}$

Confidence level is 95%, power is 90%, mean difference (µe-µc) is 2.8-164=1.16 (from the pilot trial; minimum score, 0; highest score, 10), the estimated standard deviation is 1.5, the standard deviation of the control group is 1.4, the standard deviation of the experimental group is 0.9, the pooled variance is 1.385, the ratio of the case to control is 1:1, α error is 0.05, β error is (1-power)=0.1, δ (maximum allowable error of the estimate) is 1, and the expected drop ratio is 5%, as follows: $Sample size= 2(1.38)^{2}\frac{(1.96+0.845)}{1.16^2 }^{2}$=22.27 for each group.

Two hundred first-year undergraduate medical students from two different medical colleges in Rajkot (Gujarat) participated in this study after providing informed consent. The students who had cleared the National Eligibility-cum-Entrance Test-Undergraduate 2021 (NEET-UG-2021) and confirmed their admission to the first year of the Bachelor of Medicine and Bachelor of Surgery courses before starting the study were included. The students who did not have a basic knowledge of computers and/or were not capable of operating smartphones were excluded. The participants were arranged in order from the highest rank to the lowest rank in NEET-UG-2021; the odd-numbered participants were allocated to the control group, and the even-numbered candidates were allocated to the experimental group. The participants were allocated to the control (n=100) and experimental (n=100) groups using a systemic random selection method based on the All India Rank in NEET-UG-2021 after matching for age and sex.

Intervention and post-test score

Control learning resources were provided to the control group, and interventional learning resources were provided to the experimental group in training sessions of 30 minutes, which were designed for teacher-guided self-directed learning.

The participants’ knowledge of the sacrum was assessed before and after the learning session using a questionnaire comprising 10 image-based single-best-answer multiple-choice questions (MCQs) designed by senior anatomists who were not involved in designing the learning resources according to the learning objective for each specimen; five questions addressed the external features, and five questions addressed the relations and attachments of the sacrum. The participants were provided 10 minutes to complete the questionnaire; qualified anatomists assessed the responses of all participants using a single-blind method. The participants who scored 100% in the pre-test or with pre-test scores more than those within the normal distribution curve were not considered for the data analysis.

Data analysis

The mean pre-test and post-test scores were calculated and analyzed for normal distribution, and the results were compared between the control and experimental groups (unpaired one-tailed Student’s t-test, 95% confidence interval {CI}, and p≤0.05).

## Results

The difference in the mean ages of the participants in the control (18.2±1.5 years) and experimental (18.1±1.6 years) groups was not significant (considering 95% CI and p<0.05), and both groups had equal male-to-female ratios (84:16).

The pre-test score distribution curve was normal (Chen-Shapiro test, p=0.092), and the difference in pre-test scores between the control and experimental groups was not statistically significant (two-tailed unpaired Student’s t-test, p<0.05, and 95% CI). The pre-test score of the sacrum control group was 1.3±0.8 (n=100) and the experimental group 1.1±0.7 (n=100) (t-statistics, 1.88; degrees of freedom {df}, 198; and p, 0.0614).

The post-test score of the control group and the experimental group increased significantly after the learning session (Table 1; paired Student’s t-test, p<0.05, and 95% confidence interval). The mean difference between the post-test score and the pre-test score of the experimental group (4.1±1.6) was significantly higher than the difference between the post-test and pre-test scores of the control group (2.5±1.2) (Table 1; unpaired tailed Student’s t-test, p≤0.05, and 95% confidence interval). The difference was significantly higher in the experimental group for the external features, relations, and attachments of the sacrum (Table 1).

**Table 1:** Mean difference of post-test and pre-test scores among the control and experimental groups SD, standard deviation; df, degrees of freedom

Group	Mean difference (mean±SD)		T statistic	df	P-value
	Control group	Experimental group			
External features	1.4±0.7 (n=100)	1.9±0.8 (n=100)	4.54	198	<0.005
Attachments	1.2±0.8 (n=100)	2.2±1.0 (n=100)	7.99	198	<0.005
Total	1.6±0.9 (n=100)	4.1±1.6 (n=100)	7.78	198	<0.005

## Discussion

Two hundred students participated in the study after providing informed consent, out of which 100 students were allocated to the control group and 100 students were allocated to the experimental group using the systemic randomization method. We divided the learning objectives into two sections; the first section included external features, and the second section included the attachment and relations of the sacrum. For the knowledge assessment, we structured a pre-test and post-test composed of 10 image-based MCQs; 50% of the questions were related to the first learning objective, and the rest were related to the second learning objective. We found that VI3DM effectively increased the post-test score in comparison to conventional teaching aid for both learning objectives. Our result is supported by previous studies that reported that VI3DM is engaging and improved the scores of the test group [9,11-14]. VI3DM is the virtual/digital presentation of all three dimensions of the structure, and it gives the observer the interactive spatial orientation of the structure in a virtual environment [15-18].

The results indicate that using VI3DM as a learning aid improves the short-term memory retention of external features, relations, anatomical position, and attachments of the sacrum. The use and effectiveness of any teaching aid depend on the complexity of the learning objective and learning outcomes [9-14]. Anatomy is a subject where spatial understanding is very important to understand the relations and external features of any structure. The conceptualization and memorization of sacrum anatomy require the processing of high cognition and visuospatial information, which is affected by cognitive load [18]. According to the cognitive theory of learning, learners can understand easily and retain information for a longer time whenever they demonstrate the learning topic or interact with the object [18,19]. According to the theory, interaction with VI3DM of the sacrum may help learners to conceptualize the anatomy of the sacrum. In addition, the use of VI3DM using a platform such as virtual reality isolates the learners from the surrounding environment and decreases distraction. In this study, the physical specimen of the sacrum, which can allow learners to interact with it, was also provided to both the control and experimental groups; the experimental group improved higher in the post-test [19-22]. The reason should be that the additional VI3DM of the sacrum provided to the experimental group was annotated, which may have simplified the understanding of the sacrum, and VI3DM is engaging in learning and keeps learners focused during the session [20-22].

Limitation

In this study, we used only one specimen; an extensive study using multiple specimens can be useful for the consistent effect of VI3DM in anatomical education.

## Conclusions

Virtual interactive three-dimensional models are effective to conceptualize the anatomy of the sacrum and can be effectively used in medical education. Further applications of virtual reality and three-dimensional models should be explored in medical education and various fields of health services. The study should be performed to find the relationship between the cognitive load of any learning objective and the advantage of 3D technology to conceptualize the learning topic.
